# Evaluation of human papillomavirus as a risk factor in prostate cancer pathogenesis

**DOI:** 10.1007/s12672-025-03236-1

**Published:** 2025-07-19

**Authors:** Richard Maliye, Shari Babu, Anna E. Harris, Rodhan Patke, Atara I. Ntekim, Catrin S. Rutland, Victoria James, Nigel P. Mongan, Srinivasan Madhusudan, Jennie N. Jeyapalan, Musalwa Muyangwa Semenova

**Affiliations:** 1 School of Medicine, University of Zambia, Lusaka, Zambia; 2 Faculty of Medicine and Health Science, University of Nottingham Biodiscovery Institute, University Park, Nottingham, UK; 3 Department of Radiation Oncology, College of Medicine, University of Ibadan, Ibadan, Nigeria

**Keywords:** Prostate cancer, Human papillomavirus, Benign prostate hyperplasia, Oncogenesis

## Abstract

Prostate cancer (PCa) is the most prevalent and leading cause of cancer-related deaths among men in most countries around the world, with sub-Saharan Africa being among the most severely affected regions. Indeed, PCa is more common and lethal in indigenous African men, African Americans, and Afro-Caribbean men as compared to their age-matched white counterparts. While the fundamental aetiology of PCa and the role of androgen signalling are well understood, the basis of this racial disparity in PCa incidence and progression remains poorly understood. In this review we revisit the potential association of human papilloma virus (HPV) and PCa. While several studies support an association between HPV and PCa progression and aggressiveness, the importance of HPV in PCa is not without controversy. Here we evaluate studies that both support and challenge a mechanistic role for HPV in PCa and discuss limitations of these studies. We offer suggestions for future studies to address the contribution of HPV to the racial disparity in PCa incidence and outcomes.

## Introduction

The prostate gland is a walnut-sized composite male accessory sex organ, resembling a truncated cone in shape, located between the bladder and the penis [1]. Prostate cancer (PCa) is most commonly diagnosed as organ-confined adenocarcinoma, but progression to locally advanced and distanced metastases is common [2, 3]. The global prevalence of PCa continues to increase. From 1990 to 2017, the age-standardised incidence rate (ASIR) increased from 30.5 to 37.9 cases per 100,000 people [4]. This increase over time makes PCa the fifth most common cancer today and the most common non-cutaneous cancer in men worldwide [5, 6]. A 2019 study reported that PCa had the highest incidence in men in 114 countries and was the leading cause of cancer-related deaths among men in 56 countries worldwide in 2017 [6]. More recently, ‘The Lancet Commission on Prostate Cancer’ highlighted that annually from 2020 to 2040, the number of new cases will increase from 1.4 million to 2.9 million, prompting new strategic aims for understanding prostate cancer in ethnic groups under-represented in current research and to deliver early diagnosis and better treatment options [7].

The known risk factors for PCa are older age, race/ethnicity, and family history [4, 8, 9]. Other factors such as geographic conditions, dietary habits, physical activity, and occupational exposures are controversial [4, 8]. PCa is more common in men of African descent, where the disease is often more aggressive, with increased mortality contributing to greater PCa-related deaths compared to men from other ethnic backgrounds [10]. Since 1990, countries such as Zambia, Zimbabwe, Mozambique, and the Central African Republic have recorded a 25% increase in PCa cases, while Libya, Tunisia, and Mauritania recorded over a 125% increase over the same period [4]. Furthermore, the number of PCa-related deaths in sub-Saharan Africa (SSA) is predicted to increase by 95%, from 39,000 in 2015 to 76,000 by 2035 [5].

PCa thus presents a major health burden, yet the aetiological basis for the racial disparity in incidence and outcomes is poorly understood [4, 11]. The molecular mechanisms contributing to prostate carcinogenesis are an area of active research [12–15]. In this review, we will focus on the potential contribution of viral infection to PCa carcinogenesis. There is epidemiological evidence that the prostate gland is susceptible to infection by several types of viruses that have oncogenic potential. Some of these viruses could therefore play a role in the malignant transformation of the normal prostate cells [8, 16–18]. Viruses such as polyomaviruses, human papillomaviruses (HPVs), and members of the herpes virus family have all been found to be oncogenic [8, 19, 20]. These viruses are also known to induce local inflammatory processes that in turn cause cellular changes that can contribute to genetic and epigenetic alterations that lead to cell transformation [21].

The role of HPV infection in PCa has been the focus of numerous studies over recent years. Here we provide a summary of the molecular characteristics of HPV subtypes, review their potential role in PCa, and briefly consider the implications of HPV vaccination on PCa prevalence. Finally, we provide some recommendations to be considered in future HPV-PCa research to improve the validity and reliability of the findings.

### Human papillomaviruses

HPVs are mostly transmitted through sexual contact and are amongst the most common sexually transmitted infections in the world, causing different disease types from benign lesions to invasive tumors. Over 200 HPV genotypes have been identified, of which 40 are implicated in anogenital tract infections [22–25].

**Fig. 1:**
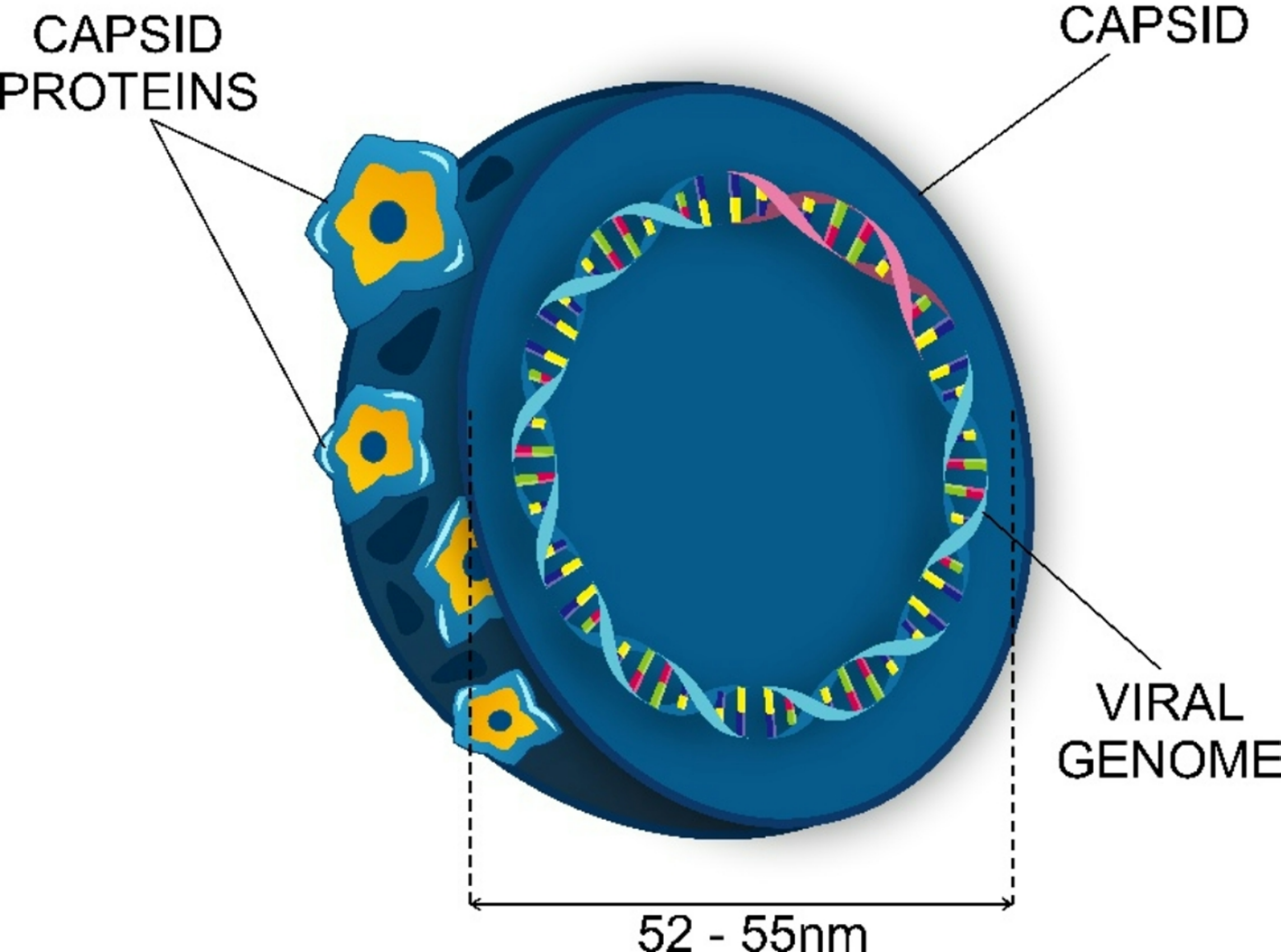
HPV particle. The virions of the viruses of the papillomaviridae family consist of iscosahedral capsids that are about 55 nm in diameter, containing double stranded DNA

HPVs are a large family of small, non-enveloped, double-stranded DNA viruses that infect human epithelial cells (the cutaneous and mucosal epithelium) and use cellular machinery of the host to replicate [26–28]. HPV viral particles are icosahedrons with a diameter of approximately 52–55 nm and contain a single, circular double-stranded DNA genome that is about 8,000 nucleotide base pairs long (Fig. 1) [25, 29].

The HPV capsid is made up of two structural proteins, namely L1 and L2. Both proteins are encoded within the viral genome. L1 is 55 kDa in size and constitutes 80% of the total viral protein. L2 is larger than L1, with a molecular mass of 70 kDa. When centrifuged in caesium chloride, the intact virion has a density of 1.30 to 1.33 g/ml [29, 30].

All HPV types consist of a genome encoding eight open reading frames (ORFs), all of which are transcribed from the single DNA molecule [31]. The ORF is divided into three functional segments, namely (1) the early (E) region, (2) the late (L) region, and (3) a largely non-coding segment. The E region encodes proteins designated as E1–E7, required for viral replication. The genes for the viral E proteins are encoded within the early promoter region, whereas the L proteins are encoded mainly within the late promoter region. The L region encodes the L1 and L2 structural proteins required for virion assembly. L1 and L2 proteins assemble in capsomers, thereby forming icosahedral capsids around the viral genome when progeny virions are being generated. The non-coding segment is referred to as the long control region (LCR) and contains *cis*-elements important in viral DNA replication and transcription processes [26–28].

The E1–E7 viral proteins play complex, specialised roles in the virus life cycle and propagation [25, 31, 32]. E1 and E2 act as recognition factors of replication origins. In addition, E2 is also the principal regulator of the transcription of viral genes. E4 is thought to be involved in the late stages of the virus life cycle, while E5 is believed to function in both early and late stages [31]. E6 and E7 proteins act by targeting several negative regulators of the cell cycle and promote the maintenance of stable viral episomes throughout the life cycle of the virus. The named proteins also induce differentiating cells to re-enter the S phase and interfere with the function of certain host cell proteins [25, 31].

HPVs are sub-classified into low-risk (LR) and high-risk (HR) distinctive groups, based on their ability to cause cancer (Table 1). The LR-HPVs cause low-grade lesions such as benign warts and respiratory papillomas [33]. The HR-HPVs, on the other hand, are primary causative agents of carcinomas and are believed to be responsible for ~ 5% of all cancer cases worldwide [25, 33–35].

**Table 1:** High- and Low-risk HPV groups

HPV groups	HPV sub-types
High risk group	16, 18, 31, 33, 35, 39, 45, 51, 52, 56, 58, 59, 66
Low risk group	6, 11, 40, 42, 43, 44, 54, 61, 70, 72, 81

HPVs have been implicated in urological, gynaecological, cutaneous, and oral squamous carcinomas. HR-HPV infection is a causal agent in cancers of the cervix, anus, tonsils, larynx, and head and neck. Currently, HR-HPVs are also considered in the pathogenesis of penile, testis, bladder, renal, and even prostate cancer [25, 36].

**Fig. 2:**
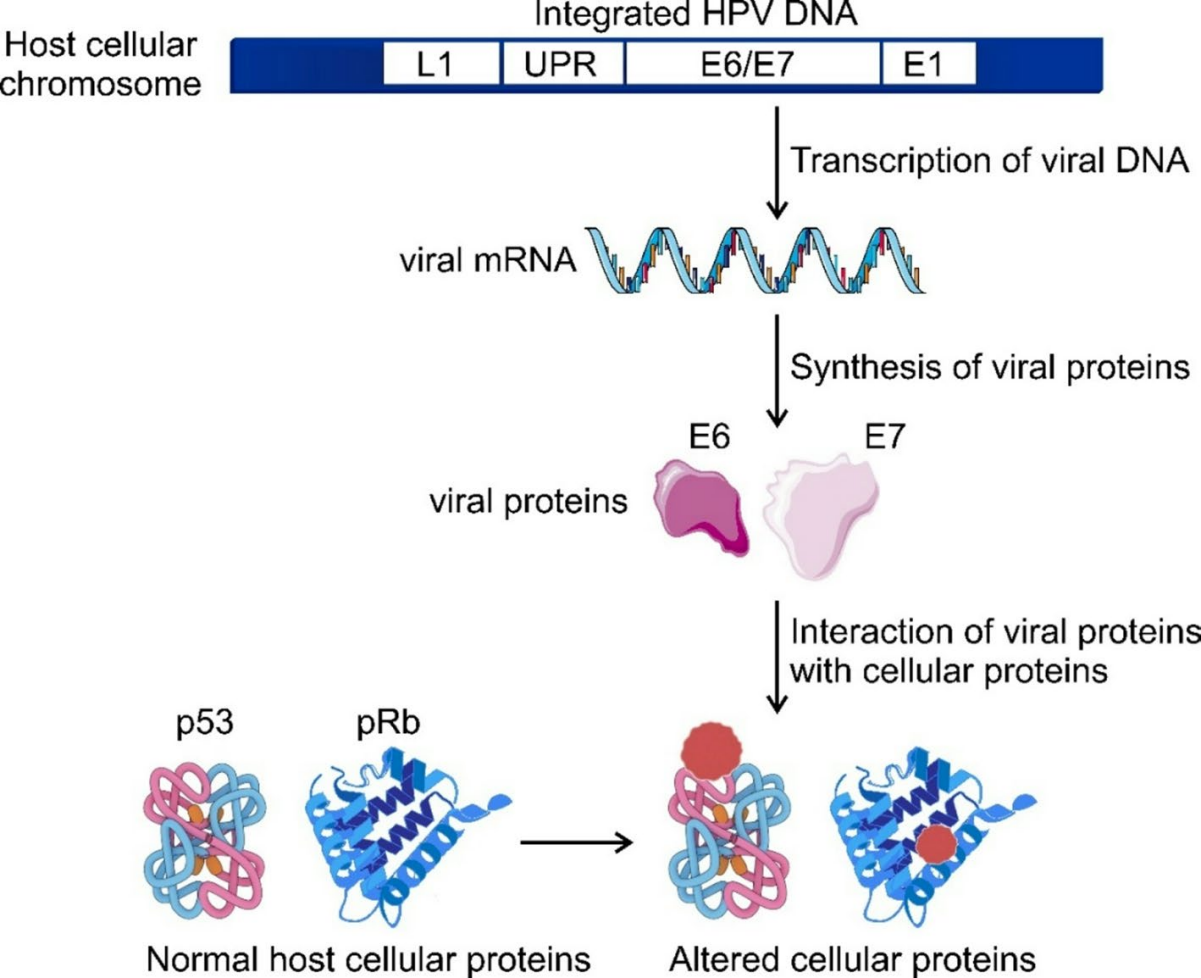
Expression of HPV genes in an infected cell The illustration shows the HPV-18 sequence that has integrated into the HeLa genome. The synthesized E6 and E7 proteins respectively bind to and promote the degradation of the host cellular proteins p53 (E, early gene; L, late gene; UPR, upstream regulator sequence)

The biology underlying HPV-associated carcinogenesis is complex and involves multiple mechanisms promoted by the viral oncoproteins E6 and E7 [25, 32]. These oncoproteins alter the normal function of critical host cellular proteins (Fig. 2). HPV E6 promotes the degradation of p53, while E7 leads to the degradation of retinoblastoma protein (pRb) [25, 32].

Both p53 and pRb are very important tumour suppressor proteins. Cells are constantly exposed to several genotoxic stresses that are damaging to DNA [37]. These genomic aberrations, if allowed to accumulate, very often result in the development of cancers. The p53 and pRb tumour suppressors play very important roles in initiating the proper stress response required to maintain genomic integrity and protect cells from malignant transformation [38].

p53 was first identified in 1979 by a number of researchers [39–44] and it functions as a nuclear transcription factor that transactivates several target genes that induce cell cycle arrest and/or apoptosis [38, 45]. Under normal cellular conditions, p53 exists in an inactive form and at very low concentrations due to the correspondingly low expression levels as well as its proteasomal degradation mediated mainly by the RING-finger type E3 ubiquitin-protein ligase MDM2. Upon cellular DNA damage, however, p53 accumulates in the nucleus and is activated through covalent modifications such as phosphorylation and acetylation of its target functional groups. When there is damage within the genomic DNA, p53 mediates cell cycle arrest to allow for the damaged DNA to be repaired, whereafter the cells re-enter the normal cell cycle. When cells have serious DNA damage, p53 instead promotes apoptosis to eliminate cells with irreparable DNA damage and prevent their transfer of aberrant DNA to daughter cells [38]. p53 brings about its effects as a tumour suppressor by activating the transcription of approximately 2,200 genes. In addition, p53 is not only a transcription activator, as it was considered initially, but also downregulates the transcription of about 2,700 genes. This transcription repression by p53 is mostly indirect and involves a protein complex termed the dimerisation partner (DP), RB-like E2F, and the MuvB (DREAM) complex, in an axis known as the p53-DREAM pathway [46].

pRb is a part of the retinoblastoma family of proteins, consisting of three members, namely Rb/p105, p107, and Rb2/p130. These proteins are collectively referred to as the “pocket proteins” [47, 48]. Like p53, pRb plays important roles that prevent tumour progression. When the genome is exposed to genotoxic conditions, pRb blocks S-phase entry and cell growth by inducing the G1 checkpoint. pRb is also important in promoting apoptosis when there is serious DNA damage that cannot be easily repaired [37, 47]. The mechanisms by which p53 and pRb promote their effects are not necessarily independent. Some of the effects of p53 are mediated by the recruitment of pRb. In response to p53 activation, pRb cooperates with DREAM to induce gene repression, thereby preventing the transcription of genes for proteins required for the transition from G1 to S phase of the cell cycle [37, 49].

As highlighted above, p53 levels are low under normal cellular conditions, in part, due to MDM2-mediated p53 degradation. MDM2 covalently modifies the p53 protein through post-translational ubiquitination, thereby rendering the tumour suppressor susceptible to degradation. However, HPV E6 targets p53 by manipulating this ubiquitin-mediated degradation pathway and leads to a significantly and abnormally increased rate of p53 degradation [38, 46]. On the other hand, studies on HPV-16 oncogenicity found that E7 binds to pRb and impairs its function as a tumour suppressor [46, 50]. E7 oncoprotein inactivates pRb along with the other pocket proteins by inhibiting the ability of these host proteins to bind certain transcription factors that are necessary for mediation, such as E2Fs. In addition, E7 also targets the pocket proteins for degradation. Evidence also shows that E7 disrupts some of the functions of the DREAM complex, thereby interfering with the p53-DREAM pathway and ultimately compromising the function of p53 as a tumour suppressor [46, 48]. Considering the vital roles played by p53 and pRb, the actions of the HPV oncoproteins thus compromise cellular ability to maintain genome integrity and render cells more susceptible to mutagenesis.

Although HPV E6 and E7 expression are important for promoting cell proliferation through the inactivation of tumour suppressor proteins p53 and pRb, these mechanisms alone are not sufficient to drive cancer pathogenesis [51]. A necessary condition for cancer progression is an accumulation of additional somatic mutations over decades of persistent HPV infection. These mutations are thought to be promoted in part by the action of a family of proteins known as apolipoprotein B mRNA editing enzyme, catalytic polypeptide-like 3 (APOBEC3 or A3) proteins [51, 52]. A3 proteins are a family of interferon-inducible cytidine deaminases that contribute to the innate immune response against viral infections (including HPV) by functioning as antiviral restriction factors, and they are normally produced in response to infection by viruses and other pathogens [32, 53–55]. A3 proteins act by converting cytosine to uracil in single-stranded DNA exposed during DNA replication [52]. Seven A3 family members are expressed in humans, and their genes are located on chromosome 22. These are A3A, A3B, A3C, A3D, A3F, A3G, and A3H [54, 55].

Analyses of data from genome sequencing studies show that overexpression of either A3A or A3B protein is associated with increased mutagenesis [56]. Furthermore, infected cells have been found to have a high prevalence of A3A and A3B mutation signatures in HPV-associated cancers. The major triggers for A3A- and A3B mutations are E7 and E6. During persistent HPV infection, the aforementioned oncoproteins are expressed in high levels; E7 specifically upregulates A3A, while E6 promotes overexpression of A3B [51].

**Fig. 3:**
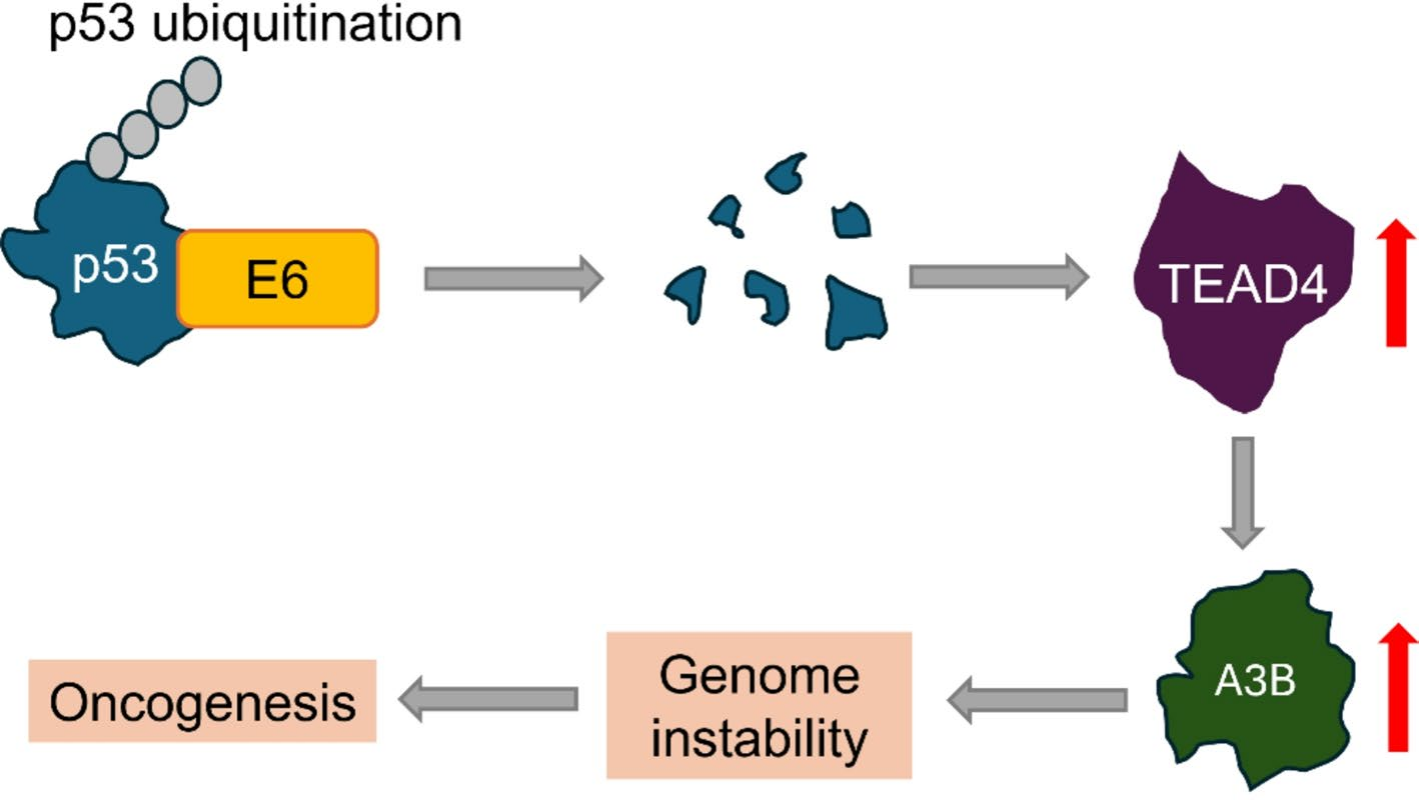
A3B-mediated HPV oncogenesis. HPV-E6 oncoprotein binds to p53 which leads to ubiquitination and degradation of p53. Loss of p53 leads to up-regulation of TEAD4 (mechanism not known). TEAD4 up-regulates A3B which leads to an increase in genomic mutations and oncogenesis (up-regulation, red arrows)

The mechanism by which E6 promotes overexpression of A3B is mediated by a family of transcription factors known as transcriptional enhancer domains (TEADs) [51]. There are four TEAD family proteins expressed in mammals: TEAD1, TEAD2, TEAD3, and TEAD4. TEADs recognise the consensus DNA sequence (AGGAATG), which is termed the MCAT motif [52]. E6 stimulates A3B overexpression indirectly by upregulating the TEAD4 transcription factor. TEAD4, in turn, recognises and binds to A3B promoter regions to induce the expression of A3B. The exact mechanisms by which E6 upregulates TEAD4 are unclear, but they seem to require p53 degradation [52]. Overexpression of A3B proteins induces mutations throughout the genome and promotes an increased risk of several cancers, including PCa [32, 53] (Fig. 3).

The E7 oncoprotein from HR-HPVs (but not LR-HPVs) promotes a dramatic increase in A3A levels through multiple mechanisms that considerably extend the half-life of A3A. For example, HR-HPV E7 inhibits proteasome-dependent degradation of A3A [54]. Interestingly, however, the A3A protein restricts HPV infection [57] and would otherwise be detrimental to the virus life cycle. HR-HPVs are therefore thought to have means through which they evade restriction by A3A [54]. For example, the TC dinucleotides, which are the preferred sites for A3A targeting, are significantly under-represented in HR-HPVs as compared to the LR-HPVs [54, 57]. A3A elevation thus seems to be an inadvertent consequence of cellular activities of HPV, with HR-HPVs evolving to simply evade the consequences of this A3A upregulation.

In addition to the APOBEC-mediated mechanisms, it is suggested that other molecular components of HPV-viral particles might contribute to the transformation of benign prostate epithelium to malignant tissue via Toll-like receptor (TLR) pathways [58]. Just like the APOBEC proteins, TLRs are a component of the innate immunity system and mediate the response of the immune system by preventing entry of the pathogen into host cells, detecting and warning of pathogen presence within the early stages of infection, regulating inflammation, stimulating an adaptive immune response, and actively participating in combating infection [59]. TLRs are transmembrane receptors that act by recognising pathogen-associated molecular patterns (PAMPs) such as flagellin, lipopolysaccharides, double-stranded RNA, and non-methylated DNA. TLRs initiate a cascade of molecular events involving multiple intracellular proteins. The primary effect of the TLR pathways is to activate transcription factors such as nuclear factor kappa B (NF-kB), interferon regulatory factor 3 (IRF-3), interferon regulatory factor 7 (IRF-7), and activator protein 1 (AP-1). These transcription factors then mediate the secondary effects of the TLR pathways by promoting the transcription of several genes leading to the synthesis of pro-inflammatory proteins, T-cell stimulators, and cytokines. The aforementioned transcription factors also induce the expression of antiviral response genes such as interferons [59–61].

Ten TLRs have been identified in humans, and each type recognises a distinct PAMP. These TLRs are further classified into two groups based on their cellular location. TLR1, TLR2, TLR4, TLR5, and TLR6 are found on the plasma membrane and are activated by extracellular PAMPs. In contrast, TLR3, TLR7, TLR8, and TLR9 are located in membranes of intracellular compartments such as endosomes and lysosomes, where they detect DNA/RNA released from viruses or bacteria that are degraded within the aforementioned organelles inside the cell [59, 62, 63]. The PAMP recognised by TLR10 is not known, and this receptor remains one of the least understood TLRs [64, 65].

**Fig. 4:**
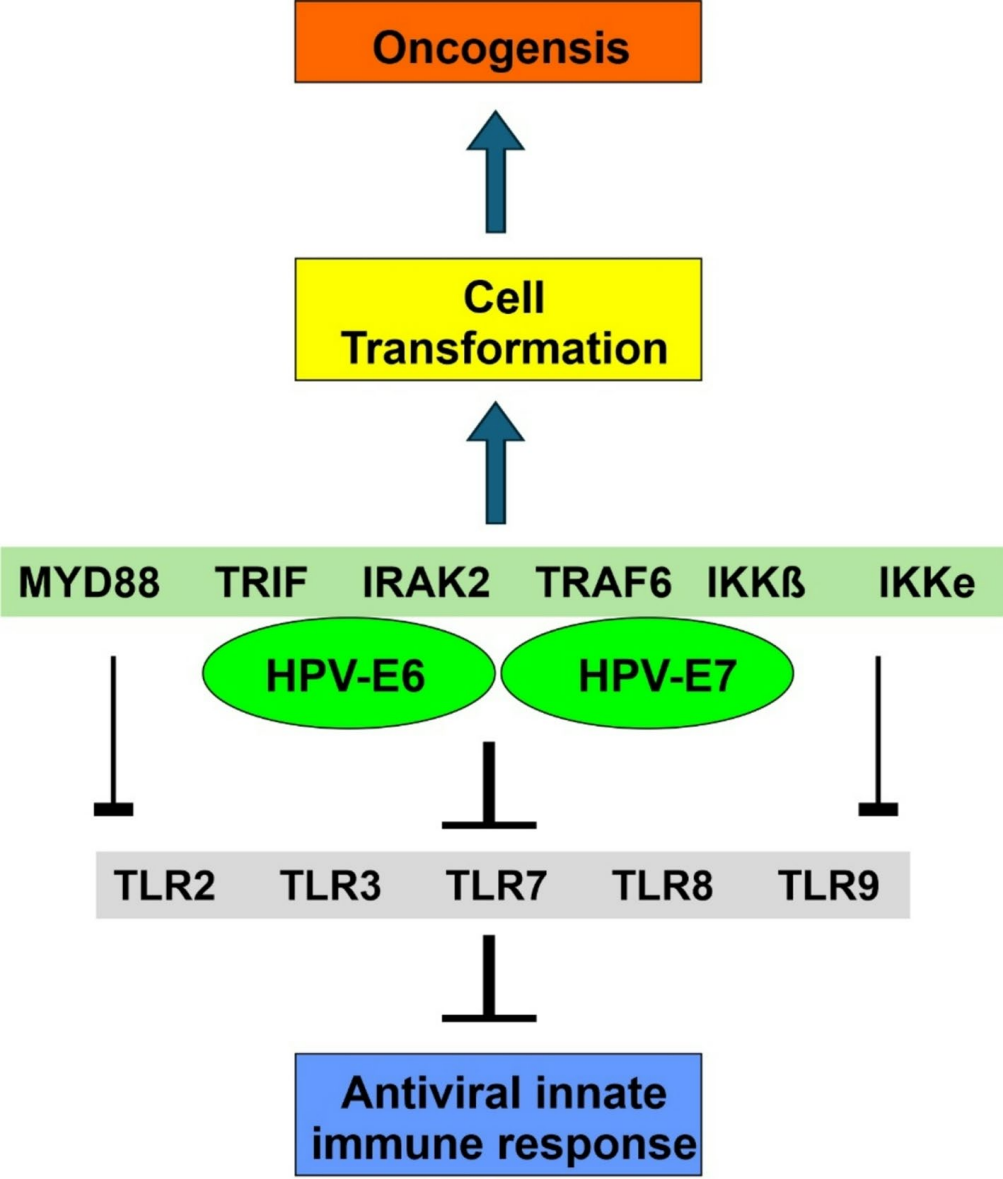
HPV oncogenesis via interference of TLR pathways. HPV infection has been shown to down-regulate the TLR pathway and in-turn the viral innate immune response. Proteins involved in the TLR pathway (shown in light green box) are indirectly/directly affected by HPV-E6/7 binding, which in-turn disrupts the TLR pathway, while also promoting oncogenesis. *TLR* toll-like receptor, *MyD88* myeloid differentiation primary response protein, *TRIF* TIR domain-containing adaptor molecule 1, *IRAK2* interleukin-1 receptor-associated kinase-like 2, *TRAF6* tumor necrosis factor (TNF) receptor-associated factor 6, *IKKβ* I-kB kinase beta, *IKKγ* I-KkB kinase epsilon

Expression of HR-HPV oncoproteins has been found to have an inhibitory effect on *TLR-9* mRNA expression and protein activation in keratinocytes [66, 67]. Conversely, it has also been consistently demonstrated that regression of HPV16 infection is significantly associated with increased expression of TLR2, TLR3, TLR7, TLR8, and TLR9 [68]. These results underscore the protective role of TLRs against HPV infections and suggest that HPV establishes itself in the infected cell by downregulating TLR pathways (Fig. 4).

Oliveira et al. found that HPV E6 oncoprotein interferes with TLR pathways by interacting, either directly or indirectly, with six TLR pathway proteins: myeloid differentiation primary response protein (MyD88), TIR domain-containing adaptor molecule 1 (TRIF), interleukin-1 receptor-associated kinase-like 2 (IRAK2), tumour necrosis factor (TNF) receptor-associated factor 6 (TRAF6), I-kB kinase beta (IKKβ), and I-kB kinase epsilon (IKKε) [69]. Despite their central roles in the TLR response, the abovementioned proteins may also contribute to cell transformation under HPV influence. For example, MyD88 is thought to have a pro-tumour role by participating in the inflammation process, thereby contributing to cancer progression in the skin, liver, pancreas, and colon [70–72]. Likewise, there is evidence that IKKε also plays an important role in viral carcinogenesis [73, 74]. More studies on E6 interaction partners are required to better understand the relationship between E6 and TLR signaling. Equally important will be the ability to distinguish between direct E6 interaction partners and the indirect ones, a task that might prove difficult when the identified partners are part of the same signalling pathway [69].

### Link between HPV and prostate cancer

Interest in the potential causative role for HPV in PCa grew after HPV DNA was detected in biopsies of anal cancer. Since epidemiological evidence shows that men who develop anal cancer were more likely to develop PCa as well, it was hypothesised that HPV could have a direct involvement in PCa pathogenesis [75, 76]. While several studies sought to address potential links between HPV, anal, and prostate cancers, results obtained to date have been inconsistent (Table 2). This section will review the identification of HPV in PCa samples, clinical associations with the presence of HPV and studies that also address the activities of E6/E7 in prostate tissue.

**Table 2:** A summary of studies on HPV and prostate cancer

Authors	Location	Year	Cases (*P*/T)	Controls (*P*/T)	Samples	Detection method	HPV genotype	Association (HPV and PCa)	Refs.
McNicol and Dodd	Canada	1990	4/4	15/20	Tissue	PCR	16, 18	Yes	[77]
McNicol and Dodd	Canada	1991	14/27	35/61	Tissue	PCR	16, 18	No	[129]
Anwar et al.	Japan	1992	28/68	0/20	Tissue	PCR	16, 18, 33	Yes	[79]
Effert et al.	USA	1992	0/30	N/A	Tissue	PCR and Southern blot hybridizations	16, 18	No	[93]
Ibrahim et al.	USA	1992	6/48	2/36	Tissue	PCR, ISH	16	No	[94]
Rotola et al.	Italy	1992	6/8	14/17	Tissue	PCR	16	No	[130]
Dodd et al.	Canada	1993	3/7	5/10	Tissue	PCR	16	No	[95]
Tu et al.	USA	1994	1/43	0/18	Tissue	PCR	16, 18	No	[96]
Moyret-Lalle et al.	France	1995	9/17	7/22	Tissue	PCR	16, 18	Yes	[131]
Wideroff et al.	USA	1996	7/56	4/42	Tissue	PCR	6, 11, 16, 18, 31, 33, 45	No	[97]
Anderson et al.	UK	1997	0/14	0/10	Tissue	PCR	16	No	[132]
Terris and Peehl	USA	1997	2/53	4/78	Tissue	PCR	16	No	[133]
Dillner et al.	Finland	1998	40/165	60/290	Serum	ELISA	11, 16, 18, 33	Yes	[80]
Noda et al.	Japan	1998	0/38	3/71	Tissue	PCR	16, 18, 31, 33, 35, 52, 58	No	[134]
Strickler et al.	USA	1998	0/63	0/61	Tissue	PCR	11, 16, 18, 51, 56	No	[112]
Serth et al.	Germany	1999	10/47	1/37	Tissue	PCR	16	Yes	[81]
Hayes et al.	USA	2000	19/276	15/295	Serum	ELISA	16	No	[135]
Adami et al.	Sweden	2003	69/238	48/210	Serum	ELISA	16, 18, 33	No	[136]
Rosenblatt et al.	USA	2003	81/642	64/570	Serum	ELISA	16, 18	No	[137]
Carozzi et al.	Italy	2004	17/26	12/25	Tissue	PCR	6, 11, 16, 18, 31, 33, 35, 45, 52, 58	Yes	[113]
Korodi et al.	Sweden	2005	107/799	363/2,596	Serum	ELISA	16, 18, 33	No	[138]
Leiros et al.	Argentina	2005	17/41	0/30 in hyperplasia	Tissue	PCR and Southern blot hybridizations	16, 11, pan	Yes (subset of PCa)	[82]
Bergh et al.	Sweden	2007	0/201	0/201	Tissue	PCR	N/A	No	[101]
Sitas et al.	South Africa	2007	139/205	390/673	Serum	ELISA	16	No	[107]
Sutcliffe et al.	USA	2007	107/584	114/577	Serum	ELISA	16,18,33	No	[139]
Dennis et al.	USA	2009	50/267	45/267	Serum	ELISA	N/A	No	[140]
Gazzaz and Mosli	Saudi Arabia	2009	0/6	0/50	Tissue	Hybrid Capture	6, 11, 16, 18, 31, 33, 35, 39, 40	No	[102]
Martinez-Fierro et al.	Mexico	2010	11/55	4/75	Tissue	PCR	N/A	Yes	[83]
Sutcliffe et al.	USA	2010	23/616	22/616	Serum	ELISA	16, 18, 31	No	[99]
Aghakhani et al.	Iran	2011	13/104	8/104	Tissue	PCR	6, 11, 16, 18	No	[114]
Chen et al.	Australia	2011	7/51	3/11	Tissue	PCR	18	No	[98]
Hrbacek et al.	Czech Republic	2011	HPV 6–77/329, HPV 11–40/329, HPV 16–16/329, HPV 18–8/329, HPV 31–19/329, HPV 33–7/329	HPV 6–21/105, HPV 11–15/105, HPV 16–10/105, HPV 18–10/105, HPV 31–8/105, HPV 33–5/105	Serum	ELISA	6, 11, 16, 18, 31, 33	No	[100]
Rogler et al.	Germany	2011	0/33	N/A	Tissue	PCR	1–8, 10–19, 21–26, 30–38, 40, 45–47 and 60	No	[103]
Groom et al.	UK	2012	1/100	N/A	Tissue	INNO-LiPA HPV genotyping	6, 11, 16, 18, 26, 31, 33, 35, 39, 40, 43, 44, 45, 51, 52, 53, 54, 56, 58, 59, 66, 68, 69/71, 70, 73, 74, 82	No	[105]
Salehi and Hadavi	Iran	2012	3/68	0/85	Tissue	PCR	N/R	No	[141]
Tachezy et al.	Czech Republic	2012	1/51	2/95	Tissue	PCR	16	No	[142]
Ghasemian et al.	Iran	2013	5/29	8/167	Tissue	PCR	NR	No	[115]
Pascale et al.	Italy	2013	112/150	N/A	Tissue	IHC and PCR	16	Yes	[87]
Whitaker et al.	Australia	2013	7/10	2/10	Tissue	PCR	18	No	[143]
Michopoulou et al.	Greece	2014	8/50	1/30	Tissue	PCR	16, 18, 31	Yes	[88]
Yow et al.	Australia	2014	0/115	0/51	Tissue	PCR	6, 11, 16, 18, 31,33, 35,39, 45,51,52, 56, 58, 59, 66, 68	No	[104]
Singh et al.	India	2015	39/95	11/55	Tissue	PCR	L1, 6, 11, 16, 18	Yes	[89]
Araujo-Neto et al.	Brazil	2016	0/104	0/0 N/A	Tissue	PCR	N/A	No	[76]
Davilla-Rodriguez et al.	Mexico	2016	12/62	1/25	Tissue	INNO-LiPA HPV genotyping	18, 51, 52, 66	Yes	[78]
Smelov et al.	Russia	2016	2/13	1/13	Tissue	NGS	N/A	No	[144]
Aydin et al.	Turkey	2017	1/60	0/36	Tissue	PCR and pyrosequencing	57	No	[106]
Glenn et al.	Australia	2017	19/28 (matched PCa)	23/28 (matched BPH)	Tissue	IHC	16, 18,	Yes	[90]
Medel-Flores et al.	Mexico	2018	37/189	16/167	Tissue	PCR	6, 11, 16, 18, 31, 33, 52, 58	Yes	[84]
Abumsimir et al.	Morocco	2022	8/50	0/0 N/A	Tissue	PCR and sanger sequencing	18	No	[108]
Khatami et al.	Iran	2022	21/73	7/39	Tissue	PCR	6, 11, 16, 18, 33	No	[109]
Lang et al.	China	2023	10/59	0/0 N/A	Tissue	NGS	16, 18, 26, 51, 66, 98	No	[110]
Nellessen et al.	Germany	2023	13/140	N/A	Tissue	PCR	16	No	[111]
Yin et al.	Taiwan	2024	743/5137	1069/15,411	NR	PCR or anogenital warts diagnosis	NR	Yes	[92]

Not all studies in the table have been discussed in this article, systematic reviews and meta-analysis studies have not been included in table

*PCR* polymerase chain reaction, *ISH* in situ-hybridization, *NGS* next-gen sequencing, *NR* not recorded, *N/A* not applicable, *P/T* positive cases (or controls)/total cases (or controls)

The first study to demonstrate the presence of HPV DNA in prostate tissues was reported by McNicol and Dodd in a Canadian population in 1990 [77, 78]. The authors used PCR to detect HPV16/18 in a limited number of non-malignant prostate (*n* = 5), benign prostate hyperplasia (*n* = 15), and PCa (*n* = 4) specimens. Their results indicated that HPV 16 DNA was present in 100% of the prostate carcinomas, 93% of the BPH samples, and 20% of the normal prostate tissues. HPV 18 DNA was found only in 20% of the BPH tissues that also contained HPV 16. Although the sample size was small, with only a total of 24 samples examined. This study demonstrated for the first time that HPV was present in prostate tissue and supports the potential link between HPV and PCa. The authors also suggested that the prostate tissue might act as a reservoir for sexual transmission [77]. In 1992, Anwar et al. examined HPV prevalence in a Japanese cohort and detected HPV only in the prostate carcinomas (41%, 28/68 HPV positive) and none in the BPH or normal prostate tissues. These results provided more evidence to support a link between HPV and PCa, though again sample numbers were limited [79].

Dillner et al. extended these studies by using serological approaches to compare HPV prevalence in 165 PCa cases and 290 controls. The authors found that the association between either HPV 16 or 18 and PCa risk was highly significant (PCa 24%, 40/165 positive cases; BPH 21%, 60/290 positive cases), while seropositivity against HPV types 11 and 33 showed no significant associations with PCa. These results not only supported the findings from the earlier two studies but further suggested that HPV 16 and 18 were specifically the high-risk types in PCa [80].

Serth et al. enrolled 84 men in Germany and classified PCa samples by tumour stages into 4 categories according to the tumour-node-metastasis system. HPV 16 E6 DNA was detected in 21% of PCa (10/47 positive cases) as compared to only 3% of BPH controls (1/37 positive cases) [81]. The number of samples examined was limited, but the difference found was still statistically significant. However, rather than suggesting that PCa, in general, was associated with HPV, the authors concluded that the HPV association was only limited to a subgroup of prostate cancers (not clearly indicated which one) that exhibited increased copy numbers of HPV 16 [81]. This was supported by the findings of a study from Argentina [82], which reported the presence of HPV DNA in 41% of the PCa samples (17/41 positive cases) and none in the BPH samples (*n* = 30). HPV subtypes were further investigated and identified 7 infected PCa tissues, 5 of which contained HR-HPV16, while 2 had LR-HPV11. The authors implicated HPV16 as representing HR-HPVs being associated with at least a subset of PCa [82]. In addition, the authors speculated that LR-HPVs most likely have no influence on the carcinogenesis of the prostate and pointed out the presence of HPV11 as simply demonstrating the susceptibility of the prostate gland to infection [82].

Martinez-Fierro et al. detected HPV DNA in 20% of PCa (11/55 positive cases) and 5% of the matched controls (4/75 positive cases) after examining prostate tissues from 130 men primarily from Northwestern Mexico. Different types of oncogenic HR-HPVs were found without the presence of a predominant HPV type. Notably, neither HPV 16 nor 18 were detected. The authors, therefore, concluded that although HPV was associated with PCa, the association was not due to any specific subtype of HPV as implied in other reports [83].

A study in Northeast Mexico also found different types of HR-HPVs (18, 51, 52, and 66) in 19% of PCa tissues (12/62 positive cases), without the presence of any predominant HPV type [78]. Once again, HPV 16 was not detected, reminiscent of the Northwest Mexican study six years earlier. Davila-Rodriguez et al. thus reinforced the findings by Martinez-Fierro et al. in reporting that no specific type of HPV was responsible for the apparent association with PCa [78].

In a third Mexican study, 356 prostatic tissues from unrelated Mexican men showed the presence of HR-HPVs in 19.6% of PCa specimens (37/189 positive cases) and 9.6% of BPH tissues (16/167 positive cases) [84]. This study, by contrast, reported HPV 52 and 58 as the most prevalent types in both study groups, thus setting it apart from the earlier two Mexican studies mentioned. The findings by Medel-Flores et al. were also distinctive from the rest of other studies reporting HPV 16 and 18 as the most common types in PCa patients. The discrepancies in HPV types reported by different authors might be due to variation in HPV distribution among different populations [84–86].

Pascale et al. identified HPV E7 protein expression as independently significant prognostic factor for survival in 150 patients (multivariate analysis *P* = 0.034; 112/150 positive cases; 75%) with primary PCa in Italy [87]. They also identified age, Gleason score and high nuclear grade as significant independent prognostic factors but did not analyse HPV positivity in these clinical parameters. Analysis of DNA extracted from all the immunohistochemistry-positive analysed samples confirmed HPV infection and showed the presence of the HPV 16 genotype, thus reinforcing the carcinogenicity of the HPV 16, the same subtype known to be the most carcinogenic for cervical and head and neck cancers [87].

In Greece, Michopoulou et al. used real-time PCR to examine 50 paraffin-embedded PCa tissues and 30 physiological tissues from healthy individuals. HPV DNA was detected in 16% of prostate carcinomas (8/50 positive cases) and in only 3% of normal tissues (1/30 positive cases), supporting the association of HPV infection with PCa risk [88]. No significant association was identified between HPV and clinical parameters which included PSA levels, age or stage of tumour [88].

The first Indian study to evaluate the frequency of HPV infection in PCa cases was conducted by Singh et al. HPV DNA was detected in 41% of tumour biopsies (39/95 positive cases) and only 20% of BPH samples (11/55 positive cases). In the PCa group with HPV infection, the most prevalent HPV subtypes were 16 and 18, both of which are HR types. Statistical significance was seen with HPV 16 prevalence in higher Gleason score (HPV 16 positivity in 60% tumours with Gleason score ≥ 8, *P* = 0.006), but no significance was identified with age or PSA levels. The infected BPH samples showed a prevalence of LR-HPV types 6 and 11 [89].

Glenn et al. conducted a retrospective cohort study to examine the presence of HPV DNA in Australian men who previously had BPH that later developed into PCa between 1 and 10 years. Out of the 52 patients with BPH, 54% had progressed to PCa within the study period (*n* = 28) [90]. The study investigated the expression of E7 oncoprotein using IHC in the matched BPH and PCa specimens. The E7 protein was expressed in 82% of benign specimens (23/28 positive cases) and only 29% of subsequent PCa tissues (19/28 positive cases). A similar observation was made for HPV subtypes with HPV 16 present in 15% of BPH (5/32 positive cases), reducing to only 3% of PCa tissues (1/32 positive case), while HPV 18 was identified in 26% of BPH (9/34 positive) and only 16% of the PCa tissues (5/32 positive) [90]. The implication from the study by Glenn et al. could mean that HPV promotes oncogenesis in the early stages of PCa pathogenesis but is not necessary for the maintenance of PCa, contrasting with the role of this virus in cervical cancer, where it is necessary for both initiation and progression of cancer. The role of HPV in PCa might therefore be a “hit and run” mechanism where HPV infects the host cell in the early development of cancer but becomes undetectable in the later stages. If correct, this could explain the low HPV viral load detected in PCa tissues in several studies [90, 91].

A more recent study identified HPV in 14.5% of PCa (743/5137 positive cases) and 6.9% in control samples (1069/15411 positive cases), with an adjusted odds ratio (OR) of 2.321 (95% CI 2.097–2.568) [92]. This study was notable for establishing a link between HPV and PCa from a large sample size (20548 samples), but did not investigate association of HPV with clinical parameters, such as tumour stage [92].

All studies discussed hitherto suggest that prostate carcinomas are related to HPV infection, as the named authors demonstrated significantly higher HPV DNA (or anti-HPV seropositivity) in PCa cases (36% positivity) as compared to controls (17% positivity). However, the relationship between HPV and PCa is still questionable considering there have also been many other studies that did not find evidence to support the association. In 1992, just two years after McNicol and Dodd (1990) published their pioneering study suggesting a link between HPV and PCa, Effert et al. did not find this association after analysing 30 prostate adenocarcinomas for the presence of HPV 16 and 18 using differential PCR. Neither HPV genotype was detected despite the sensitivity of their technique [93]. In the same year, another study reported that the prevalence of HPV in 84 specimens (PCa, 2/48 positive cases, 4%; controls 3/36 positive cases, 8%) examined was too low to support an aetiological role of HPV in PCa [94]. Dodd and McNicol (1993) went on to investigate expression of HPV-16 E6/E7 viral genes and showed no association preferentially with either PCa (3/7 cases positive; 43% positive) or BPH (5/10 cases positive; 50%) after assessing the transcriptional activity of HPV 16 in prostate tissues, in a small cohort of 17 samples. In conclusion, the authors pointed out that HPV was unlikely to be involved in the transformation of prostate tissue [95]. Tu et al. analysed 61 prostatic specimens and reported that only 2% of PCa tissues (1/43 positive case) had HPV 16, thus casting more doubt on the subject [96].

The role of E6 oncoprotein could still not be verified in a study with a higher sample size of 56 PCa (7/56 cases positive, 12.5%) and 42 BPH (4/42 cases positive, 9.5%) specimens, in which amplimers to E6 probes specific for HPV 6, 11, 16, 18, 31, 33, and 45 were applied [97]. Chen et al. did not find an association between PCa and HPV DNA presence in samples or previous exposure to HR-HPVs in a study that combined both tissue-based molecular techniques and serological assays. No significant difference in HPV prevalence between PCa and BPH samples was found. However, the small sample size, particularly in the control group (only 11 BPH samples), might have rendered the reliability of their findings questionable. HPV DNA was detected in 14% of PCa samples (7/51 positive cases) and 27% of BPH tissues (3/11 positive cases), with HPV 18 being the only genotype detected. Nevertheless, follow-up serological analysis showed no significant increase in anti-HPV 18 protein levels [98].

A nested case-control study of older American men used enzyme-linked immunosorbent assay (ELISA) to test for IgG antibodies against HPV 16, 18, and 31. Despite a large sample size of 616 cases and 616 controls, results did not support an association between any of the three HPVs and overall PCa risk [99]. A year later, a Czech Republic case-control study of 434 Caucasian males also reported that seropositivity rates were insignificant to warrant association (PCa, 167/329 positive cases, 51%; controls 59/105 positive cases, 56%), despite their search for more HPVs (6, 11, 16, 18, 31 & 33) [100] as compared to the abovementioned American study. This study did however observe that HPV 16 antibodies had significant association with Gleason score (*P* = 0.030) [100].

A case-control study in Northern Sweden found that HPV was unlikely to be a contributing factor to subsequent PCa progression in 352 patients examined since no HPV DNA was detected in any of the samples tested [101]. Another study did not detect HPV DNA by hybrid capture technology among 56 tested biopsies [102]. Similarly, three independent studies in Germany, Australia, and Northeastern Brazil also found no HPV DNA in their samples [76, 103, 104].

Meanwhile, a UK study found that only 1 out of 100 prostate specimens indicated weak hybridisation to HPV-16 [105]. Likewise, a Turkish study also reported that only 1.7% of PCa tissues had DNA for HPV 57, while none of the BPH samples had any HPV DNA at all [106].

A South African study also found that PCa was not associated with HPV seropositivity after analysing sera from 205 HIV-seronegative Black South Africans with PCa, using anti-HPV IgG ELISA [107]. However, the limitation of this study was that ELISA was performed using only anti-HPV-16 IgG antibodies. Thus, if other HPV types were present, they could not be detected in the samples.

More recently, a study from North Africa looked at HPV infection in PCa specimens of 50 men [108]. The study showed 8/50 men (16% positivity) were infected with HPV-18 and that there was a significant link of HPV/MMTV-like infection with Gleason score, but no other clinical parameters. Therefore, with the limitation of sample size, the study did not show a clear association between HPV and PCa [108]. Khatami et al. also showed no clear association between HPV infection and PCa with their study but did identify a microRNA signature that was altered in PCa specimens that showed HPV infection, suggesting HPV-positive tumours could be classified as a subtype, showing HPV-infection-related molecular changes [109]. A study from China also showed that HPV-positive tumours contained a specific molecular signature different from the HPV-negative PCa, but no association between HPV and PCa was found [110]. A recent study on radical prostatectomy samples found HPV DNA in 9.3% (13/140) of RP specimens, with HPV-16 being the most detected subtype (5/13 = 39%) [111]. Additionally the study found no clinical parameters such as tumour characteristics, associated with HPV status [111].

From the highlighted studies (Table 2), the link between HPV and PCa is still uncertain, even though the use of larger datasets/cohorts reports correlative findings. There is still a need for further studies to address this subject. Some researchers have speculated that HPV has no direct role in PCa pathogenesis but rather acts indirectly by bringing about inflammation in the prostate tissue, which might, in turn, lead to PCa, while others consider the prostate gland to act simply as a reservoir for HPV infection and as a site of HPV replication. The latter theory would be consistent with studies in which HPV DNA was not found to be preferentially associated with either BPH or PCa [95, 112–115].

**Fig. 5:**
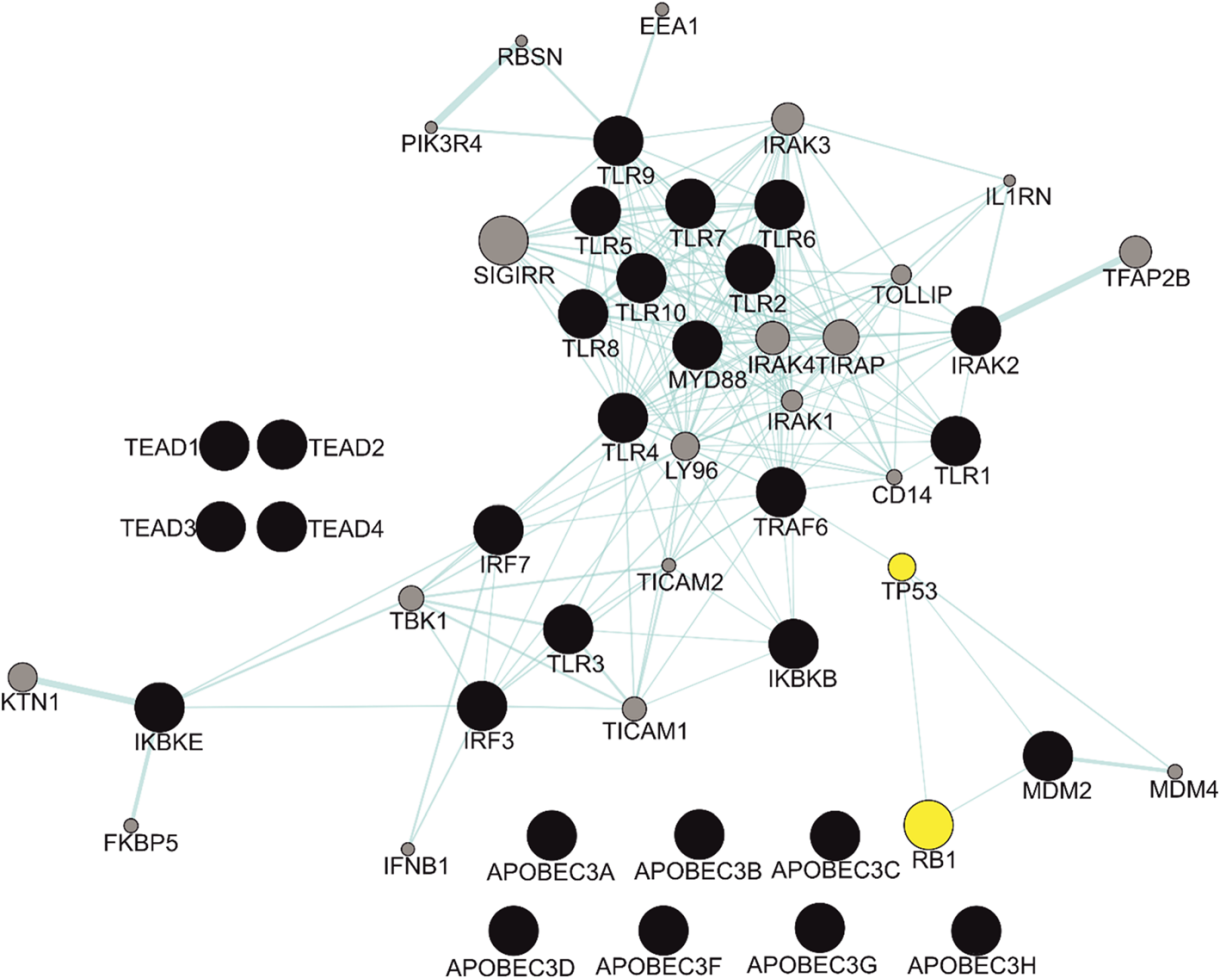
Protein interaction network of key HPV-related genes. The network map was prepared using Genemania on Cytoscape, to show both cell cycle checkpoint proteins and proteins involved in innate immunity. The TLR pathways represents the most networked cluster in the map

**Fig. 6:**
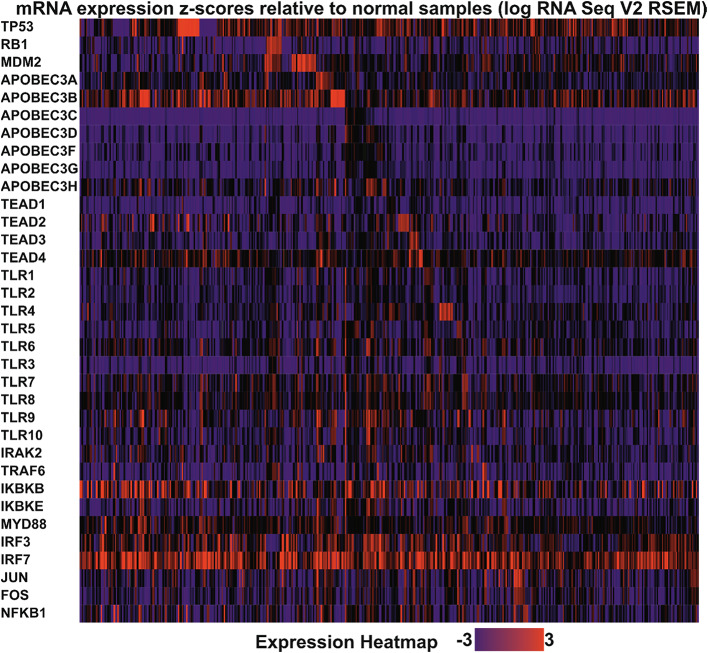
Expression of genes related to HPV infection in TCGA-PRAD cohort. Cbioportal [116], was used to assess the expression of genes related to HPV infection in the TCGA-PRAD patient cohort. The expression values (z-score) shown are relative to non-malignant samples

**Fig. 7:**
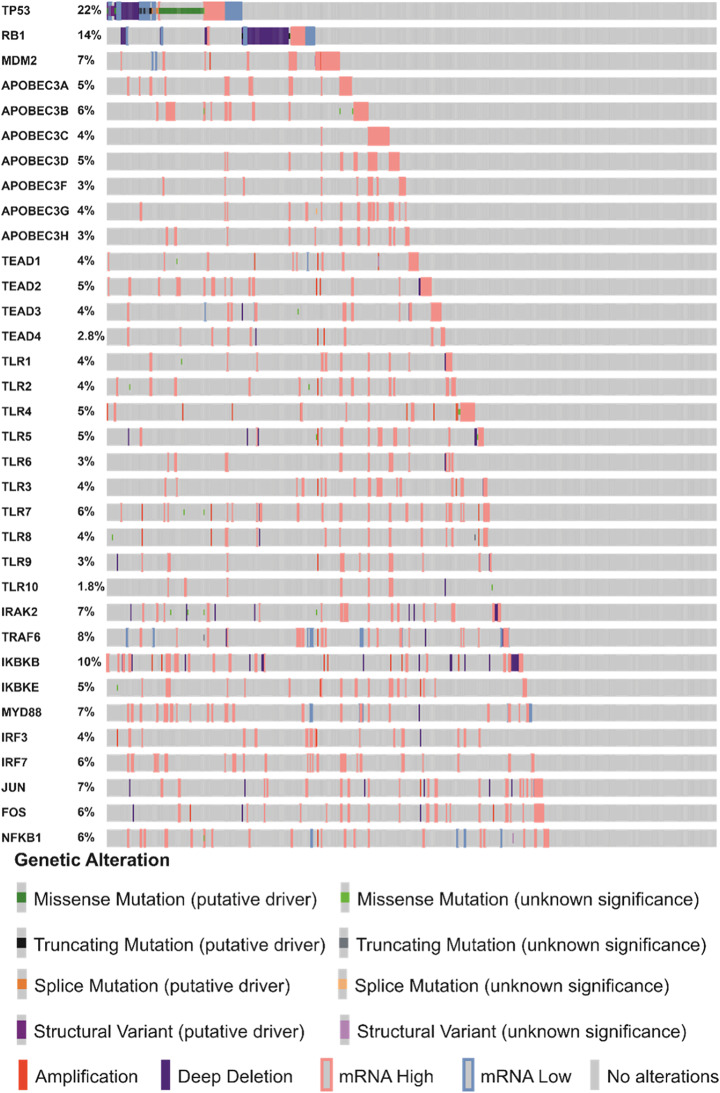
Genomic alterations in genes related to HPV infection in TCGA-PRAD cohort. CBioPortal [116] was used to assess genomic alterations in HPV-related genes in the TCGA-PRAD cohort prostate cancer specimens

Although current evidence suggests that the expression of key HPV-related genes promotes PCa development through a protein interaction network that leads to genomic alterations [116, 117] (Figs. 5, 6 and 7), further research is required to provide clearer pathway outlines, as the current models have several gaps. Establishing the HPV mechanisms for PCa is confounded by the fact that it is not agreed within the scientific community whether HPV is responsible for PCa, to begin with. Thus it will likely take several more years of research before the question on the potential role of HPV in PCa is settled.

### Research limitations

It has been more than 30 years since the first evidence of a link between PCa and HPV was proposed. Early studies were limited by small sample numbers, but more recent studies of large numbers of patients with serological approaches and more advanced contemporary molecular methods to detect and subtype HPV have not conclusively demonstrated a mechanistic link between active HPV infection and prostate carcinogenesis. Indeed, some studies on HPV and PCa apply serologic assays, but this technique inherently has several limitations, including (i) it only indicates exposure and not the exact site of infection, thus the detected HPV might be from a different tissue other than the prostate; (ii) antibody cross-reactivity may interfere with results; (iii) the temporal relationship between infection and cancer is difficult to establish; (iv) HPV generally does not induce viremia, and only persistent infections inducing pathological changes might be detected; and (v) participants might have been exposed to HPV throughout their lifetime, resulting in study bias [118].

Tissue-based studies applying PCR to detect integrated HPV DNA in prostate specimens generally have advantages over serologic studies in that molecular techniques are more sensitive. This was corroborated by a 2011 meta-analysis that reported no significant association between HPV and PCa when data were pooled to include both serologic assays and tissue-based studies but found a significantly positive association when the analysis was limited to tissue-based studies [119]. The very high sensitivity of PCR, however, renders this technique more susceptible to contamination, resulting in false positives, especially in the early studies [112, 118, 120]. Molecular techniques also have other limitations: (i) DNA detection only ascertains the current infection status and may not detect a pathogen that employs a hit-and-run mechanism of infection, and (ii) results can vary depending on the location of the tissue sampling [118].

Most tissue-based studies commonly use BPH specimens as controls rather than normal prostate specimens. This is the case because it is difficult to obtain normal prostate tissues [25, 84, 118]. However, using BPH specimens as controls likely compromises the study because it has been found that BPH itself is a risk factor for PCa, with as much as 11–44% of BPH cases progressing to PCa within 7 years [118].

Another limitation stems from the fact that a good proportion of the studies on HPV and PCa analysed very small sample sizes (see Table 2). In some of these studies, sample sizes were small across the groups. For example, Dodd and McNicol only examined 7 PCa tissues and 10 BPH samples [95], while Aghakhani et al. analysed only 13 PCa tissues and 8 BPH specimens [114]. In other studies with relatively larger sample sizes, there was group-specific bias in how the sample size was distributed between/among the groups. For instance, Anwar et al. had 68 PCa but only 10 BPH and 10 normal prostate tissues [79], while Chen et al. had 51 specimens in the PCa group but only 11 in the BPH control group [98]. Moreover, other authors only analysed PCa samples without control groups for comparison [121].

Study designs used in most research present another limitation. The majority of papers on HPV and PCa are predominantly case-control studies [121, 122]. The case-control study design has temporal uncertainty and thus cannot determine whether HPV infection precedes prostate carcinogenesis or whether the tumour environment is intrinsically more favourable for HPV infection. Case–-control studies can only suggest association but cannot implicate HPV as a causative agent of PCa [86, 123].

Most studies do not take into account or record other relevant demographic characteristics of their participants [118]. As noted earlier, PCa has a multifactorial aetiology, and age, race, and family history are all independent risk factors [4, 8, 9]. Other factors, including diet, smoking, alcohol intake, physical activity/exercise, occupational exposure, and hormonal imbalance, are also currently considered as possible risk factors [8, 85, 86]. Considering that most studies do not record these characteristics, analysis of HPV and PCa is unfortunately incomplete, as these factors might be confounding the results.

Another concern in research is publication bias. Investigation shows that there is a general tendency to publish and promote studies that show positive results, potentially excluding negative studies that fail to show an association between HPV and PCa [8]. For this reason, meta-analyses apply various methods, such as the funnel plot, to test for publication bias [122]. Some authors, such as Bae et al., Yang et al., and Yin et al. found no evidence of publication bias in HPV and PCa studies and reported that their results were not influenced by the lack of inclusion of existing negative studies into their analyses [85, 86, 118]. Moghoofei et al., by contrast, found that publication bias was significant in most cases [122]. The inconsistency reported on publication bias or lack thereof could be due to the differences in geographical regions of the studies the authors specifically included in their analyses. Although these cited meta-analyses included studies from most parts of the world, the individual studies they analysed were different. The identities of countries and the frequency with which each country appeared as the source of an included study also varied.

Most of these limitations will have to be addressed in future research to yield results with higher validity and reliability. Some authors advised future researchers to avoid contamination by putting in place and recording quality control measures [112, 118]. Yin et al. also pointed out that both serologic techniques and tissue-based methods currently in use in research are basic methods and recommended the development of novel laboratory techniques altogether if progress in this research area is to be made [118].

### HPV vaccination

With the growing list of cancer types related to HPV, there is increasing interest in extending HPV vaccination to young males with the expectation of preventing or reducing HPV-related cancers. Once such programs are implemented, there will be a need to monitor the impact of HPV vaccines in males [124]. So far, only a few countries have recommended extension of vaccination to males, including the European Union, USA, UK, and Canada [124–126].

Female vaccination should also be taken as a matter of interest in PCa prevalence studies. Although males are not directly targeted, female vaccination might give rise to herd protection; vaccinated females may stop the spread of HPV from one male already infected to another male who is not infected in cases of multiple sexual partners [124, 127, 128].

It is unfortunately difficult to speculate on the impact of female HPV vaccination on PCa prevalence among different regions because (i) HPV vaccination surveillance has not been prioritised by the World Health Organisation (WHO) due to costs and related complexity [124], and (ii) only sexually active heterosexual men benefit from female vaccination, to the exclusion of men who have sex with other men. Since most studies on PCa do not take into consideration the sexual orientation or activity of participants, it is difficult to analyse the implication of female vaccination on PCa patterns.

## Conclusion

Although data from individual underpowered studies are inconsistent, collective examination by meta-analyses has generally reported a significant association between HPV and PCa. However, there is a need for more studies, especially in Sub-Saharan Africa, where both PCa and HPV prevalence are higher compared to any other region. To address the influence of parameters such as variation in HPV prevalence and distribution and variation in risk estimates, multi-centre, large-scale studies are highly recommended. Furthermore, there is a need for future research to be orientated more towards prospective and aetiological-based studies.

## Data Availability

No datasets were generated or analysed during the current study.
